# Comparing the effectiveness and safety of Dinoprostone vaginal insert and double-balloon catheter as cervical ripening treatments in Chinese patients

**DOI:** 10.3389/fmed.2022.976983

**Published:** 2022-09-09

**Authors:** Jinjing Yan, Baomin Yin, Hanghang Lv

**Affiliations:** Department of Obstetrics, Zhuhai Center for Maternal and Child Health Care (Zhuhai Maternal and Child Health Hospital), Zhuhai, Guangdong, China

**Keywords:** cervical ripening, cesarean section, Dinoprostone, double-balloon catheter, oxytocin, vaginal delivery

## Abstract

**Background:**

This retrospective study was to compare the effectiveness and safety of Dinoprostone vaginal insert vs. double-balloon catheter as cervical ripening agents for labor induction.

**Methods:**

Pregnant women with Bishop score <7, who received either Dinoprostone vaginal insert 10 mg or Cook's double-balloon catheter for labor induction, were studied. The primary outcome was the rate of vaginal delivery within 48 h; the secondary outcomes were the proportion of women undergoing cesarean section, labor duration, oxytocin administration, changes in Bishop score, complications during labor, and maternal/neonatal outcomes.

**Results:**

One hundred and eighty-two women were included in Dinoprostone group, and 199 women were in double-balloon catheter group. The rate of vaginal delivery within 48 h was significantly higher in Dinoprostone group than that in double-balloon catheter group (90.11% vs. 75.38%, *P* = 0.0002). There were 18 cesarean section deliveries (9.89%) in Dinoprostone group and 49 cesarean section deliveries (24.62%) in double-balloon catheter group, with significant differences between two groups (*P* = 0.0002). The duration of labor was higher in Dinoprostone group, while the augmentation with oxytocin was significantly lower in Dinoprostone group than in double-balloon catheter group (all *P* < 0.0001). The incidence of chorioamnionitis was significantly higher in double-balloon catheter group as compared with Dinoprostone group (0 vs. 12, *P* = 0.0005), while neonatal outcomes were similar in two groups.

**Conclusion:**

Dinoprostone vaginal insert as cervical ripening agent is more effective for labor induction and with lower risks of chorioamnionitis as compared with double balloon catheter in Chinese populations.

## Introduction

Since January 2016, the one-child policy has been replaced by the two-child policy in China to improve the situation of stagnant population growth, aging population, and shrinking labor force, thus leading to an increase in cesarean sections (1). One recent study has shown that in China, the overall cesarean section rate of pregnant women increased from 28.8% in 2008 to 36.7 in 2018 (2). Inappropriate cesarean delivery rates pose a risk to the health of women and infants (3). The cesarean section rate of primipara should be controlled to avoid the possible risks from scarring uterine pregnancy. Therefore, increasing of Chinese obstetricians are aware of the importance of the effectiveness and safety of labor induction.

Cervical ripening, the process where the cervix becomes softened and ready for the onset of labor, occurs with both spontaneous labor and iatrogenic initiation of labor. Labor induction is one of the most common obstetric interventions to achieve successful vaginal delivery by ripening the cervix (4). In past few decades, the incidence of labor induction has continued to rise, especially in developed countries, with approximately 1 in 5 gravid women undergoing labor induction (5). A similar trend has emerged in many other high-, middle-, and low-income countries (6). The increase of selective labor induction could promote the overall trend of induction rate (7). Labor induction has significant impact on the health of women and their babies, as well as on the satisfaction of the delivery experience and the nursing organization (8). Up to now, the most efficacious method for labor induction has yet not to be determined.

According to statistics, about 1.76 million pregnant women need labor induction every year, of which nearly 1.2 million (68.5%) need to promote cervical ripening (9). Presently, there are two methods for cervical ripening: (1) mechanical methods, such as intracervical balloon catheter, amniotic membrane sweeping, or stripping (10); and (2) pharmacological methods, such as prostaglandins or oxytocin (6). Dinoprostone (a synthetic prostaglandin E2) and the dual-balloon catheter are the most popular cervical ripening agents (11, 12). Furthermore, prostaglandin cervical ripening agent in the form of a Dinoprostone 10 mg controlled-release vaginal insert as the pharmacologic method has gained widespread use in clinical practice in China (13). The off-label use of Cook's double balloon has also become popular. In the present study, we analyzed the effectiveness and safety of Dinoprostone 10 mg vaginal insert vs. double-balloon catheter in promoting cervical ripening.

## Materials and methods

### Design

This was a retrospective study that was designed to compare the effectiveness and safety of Dinoprostone vaginal insert 10 mg [Type H20090484 Controlled Therapeutics, (Scotland), Limited, Propess^®^, Ferring Controlled Therapeutics Limited, UK] and Cook's double balloon (Type J-CRB-184000 Cook Cervical Ripening Balloon^®^, Cook Incorporated, IN, USA) as cervical ripening agent in pregnant women with Bishop score < 7. The study has been approved by the local research ethics committee (No.2020111301).

### Patients' selection

Pregnant women with a Bishop score <7 requiring cervical ripening in Zhuhai Maternal and Child Health Hospital from January 2013 to December 2015 were reviewed. The inclusion criteria were the following: (1) primipara, singleton gestation, vertex presentation; (2) full-term delivery (39–40 + 6 weeks); (3) normal fetal health monitoring cardiotocography (CTG); and (4) no spontaneous uterine contractions. Exclusion criteria were the following: (1) women presented with contradictions to Dinoprostone and vaginal delivery, such as cephalopelvic disproportion, placenta previa, and other complications including mental disorders and severe heart, liver, and kidney dysfunction; (2) pregnant women with preeclampsia or complicated with chronic hypertension; (3) history of uterine or cervical surgery, including loop electrosurgical excision procedure (LEEP); (4) patients with premature rupture of the membranes (PROM), vaginitis, and abnormal liver function; (5) patients treated with both Dinoprostone vaginal insert and Cook's double balloon.

### Treatment options

Before administering the cervical ripening agent for labor induction, study subjects provided their informed consent. The procedure and potential risk of cervical ripening, including the off-label use of Cook's double balloon, were informed to patients. Following the manufacturer's instructions, a 10 mg Dinoprostone vaginal insert was placed in the posterior vaginal fornix and left in place for 24 h, unless otherwise specified. While the Cook's double balloon was introduced by injecting 50 ml of saline solution into intrauterine and intravaginal balloons, respectively. In cases such as Bishop score 7, absence of regular uterine contractions 30 min after artificial membrane rupture, or insufficient progress, oxytocin was administered to achieve regular moderate to intense contractions. With a maximum titer of 20 mU/min, oxytocin was administered at a beginning rate of 2.5 mU/min and modified every 15 min based on uterine contractions.

### Outcome measures

The primary outcome was the rate of vaginal delivery within 48 h, expressed as a percentage of the total number of women. The rate of cesarean delivery is one of the secondary outcomes. A cesarean section was performed in this situation. (1) Failure to initiate labor with induction of labor (IOL), defined as the absence of regular contractions within 48 h of IOL administration. (2) Failure in progress is defined as no change in cervical dilation or fetal head drop within 4 h of the active period. (3) Fetal distress occurs when a fetus is in jeopardy prior to or during birth. Commonly, fetal distress refers to fetal hypoxia (low oxygen levels in the fetus), which can result in fetal injury or death if it is not reversed or if the fetus is not delivered soon. In addition, other secondary outcomes included oxytocin administration, changes in Bishop score, complications during labor (birth canal injury, amniotic fluid pollution, abnormal fetal supervision, placental adhesion, and chorioamnionitis), and maternal outcome [postpartum hemorrhage (PPH), premature rupture of membranes (PROM)]. Primary PPH is characterized as significant blood loss within 24 h after delivery (500 ml for vaginal births and 1,000 ml for cesarean births) (14). Moreover, the newborn prognosis was examined (Apgar score after 1 and 5 min, birth weight, asphyxia, inhalation syndrome, infection, ICU transfer, and prenatal fetal distress). The study team collected and recorded the data simultaneously.

### Power analysis

In our study, 182 cases in the Dinoprostone group and 199 cases in double-balloon catheter group were analyzed, the results showed the rate of vaginal delivery within 48 h was 90.11% and 75.38%, respectively. An alpha risk of 0.05 and a beta risk of 0.2 were set to detect a positive result of 5% difference. Finally, the power was calculated, power = 96.35%. Power analysis was calculated using a two-tailed test in G^*^Power 3 software (Heinrich-Heine-Universität Düsseldorf, Düsseldorf, Germany).

### Statistical analysis

Continuous variables were presented as mean and standard deviation (mean ± SD) with the independent *t*-test used to compare the two groups; skewed data were presented as median and interquartile range (IQR) and compared with the Wilcoxon rank sum test. Categorical variables were presented as numbers and percentages and compared with Chi-square test or Fisher exact test as appropriate. A *P*-value < 0.05 was used as the cut-point for significance. Statistical analyses were performed using SAS 9.2 statistics software (SAS Inc., Cary, NC, USA).

## Results

**Figure 1:**
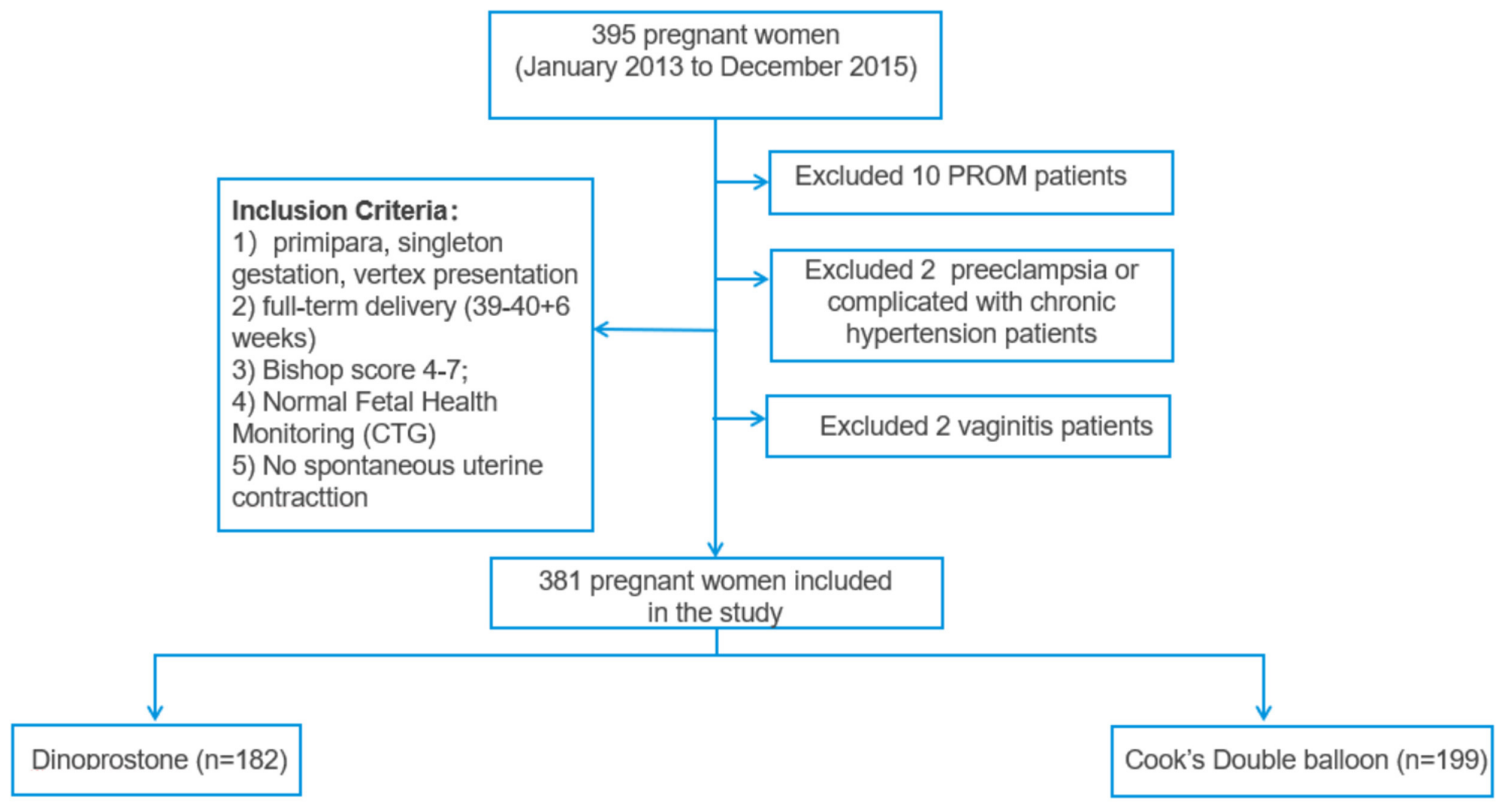
Flow chart of patients' selection.

A total of 381 women were included in Dinoprostone group (*n* = 182) and double-balloon catheter group (*n* = 199). The flow chart of patients' selection was shown in Figure 1. There was no significant difference between two groups in maternal age, body mass index (BMI), systolic pressure, gestational age, and complications of pregnant women, including amniotic fluid index (all *P* > 0.05). However, there were significant differences between two groups in terms of number of abortions, diastolic pressure, heart rate, and biparietal diameter (all *P* < 0.05) (Table 1). Those indicators would not affect the outcomes between the two groups according to a previous study (12). Bishop score was slightly higher in Cook's balloon catheter group (4.58 ± 0.69 vs. 4.88 ± 0.78), as mentioned above that patient with Bishop score ≥ 4 and <7 were more likely to be given double-balloon catheter (15, 16).

**Table 1:** Demographic and baseline characteristics.

**Characteristic**	**Dinoprostone (*n* = 182)**	**Double-balloon catheter (*n* = 199)**	***T*/χ^2^**	***P***
Gestational age (weeks)	40.78 ± 0.25	40.83 ± 0.35	−1.52	0.1291
Age (years)	30.10 ± 4.59	29.28 ± 4.42	1.76	0.0795
BMI (kg/m^2^)	26.90 ± 3.14	26.72 ± 2.85	0.58	0.5598
Number of abortions (times)				0.0324
0	104 (57.14%)	114 (57.29%)		
1	39 (21.43%)	61 (30.65%)		
2	30 (16.48%)	17 (8.54%)		
3	9 (4.95%)	6 (3.02%)		
4	0 (0.00%)	1 (0.50%)		
Systolic pressure (mmHg)	118.50 (109.00, 125.00)	118.00 (112.00, 125.00)	0.0594	0.8075
Diastolic pressure (mmHg)	69.00 (63.00, 75.00)	74.00 (67.00, 80.00)	−4.7542	<0.0001
Heart rate (beats)	86.00 (78.00, 95.00)	82.00 (78.00, 89.00)	9.6898	0.0018
Biparietal diameter (mm)	93.00 (91.00, 95.00)	92.00 (62.00, 95.00)	6.4998	0.0108
Amniotic fluid index (*n*, %)	64.63 ± 14.94	61.22 ± 24.77	1.64	0.1017
Baseline Bishop score	4.58 ± 0.69	4.88 ± 0.78	−3.99	<0.0001

BMI, Body mass index.

Table 2 showed the details of the cervical ripening. There were significant differences between Dinoprostone group and double-balloon catheter group in terms of changes in Bishop score (2.94 ± 0.94 vs. 1.92 ± 1.06, *P* < 0.0001) and the percentage of cervical effacement (40% vs. 50%, *P* < 0.0001). The duration of the first stage of labor, the second stage of labor, the third stage of labor, and the total stage of labor were significantly longer in the Dinoprostone group than in the double-balloon catheter group (all *P* < 0.0001). The proportion of patients who required oxytocin augmentation was significantly higher in the double-balloon catheter group than in the Dinoprostone group (80.90% vs. 17.03%, *P* < 0.0001). The dose and time of oxytocin administration were also significantly higher in the double balloon catheter group (*P* < 0.0001).

**Table 2:** Cervical ripening details.

	**Dinoprostone (*n* = 182)**	**Double-balloon catheter (*n* = 199)**	***T*/χ^2^**	***P***
Changes in Bishop score	2.94 ± 0.94	1.92 ± 1.06	25.48	<0.0001
Cervical effacement (%)	40 (40, 40)	50 (50, 60)	58.11	<0.0001
Oxytocin administration				
Number case (%)	31 (17.03%)	161 (80.90%)	155.13	<0.0001
Dose (IU)	2.50 (2.50, 3.75)	5.00 (2.50, 7.50)	10.14	0.0014
Time (h)	3.0 (2.00, 8.00)	8.00 (5.50, 13.00)	−4.78	<0.0001
Duration of labor (h)				
The first stage of labor	9.00 (6.00, 12.00)	4.50 (3.00, 9.25)	31.38	<0.0001
The second stage of labor	1.44 (1.00, 2.00)	0.67 (0.38, 1.24)	56.38	<0.0001
The third stage of labor	0.18 (0.17, 0.25)	0.17 (0.05, 0.17)	35.78	<0.0001
The total stage of labor	10.74 (7.60, 14.00)	6.21 (3.98, 10.57)	39.29	<0.0001

The primary outcome, which was the proportion of women who achieved vaginal delivery within 48 h was significantly higher in the Dinoprostone group than that in double-balloon catheter group (90.11% vs. 75.38%, *P* = 0.0002, Table 3). The secondary outcome was the cesarean rate, and there were 18 cesarean sections cases (9.89%) in Dinoprostone group and 49 cesarean sections cases (24.62%) in double-balloon catheter group with significant differences between the two groups (*P* = 0.0002, Table 3). Meanwhile, there were significant differences in terms of indication for cesarean section between two groups (*P* < 0.0001, Table 3).

**Table 3:** Comparison of intrapartum outcomes (primary and secondary outcomes) in two groups.

	**Dinoprostone (*n* = 182)**	**Double-balloon catheter (*n* = 199)**	***T*/χ^2^**	***P***
Mode of delivery (*n*, %)				
Spontaneous vaginal delivery within 48 h (%)	164 (90.11%)	150 (75.38%)	14.24	0.0002
Assisted vaginal delivery	0 (0.00%)	64 (32.16%)	70.35	<0.0001
Cesarean section	18 (9.89%)	49 (24.62%)	14.24	0.0002
Indication for cesarean section (*n*, %)	16 (8.79%)	47 (23.62%)	15.14	<0.0001
Failure in initiating IOL	4 (2.20%)	10 (5.03%)		
Fetal distress	10 (5.49%)	27 (13.57%)		
Failure in progress	2 (1.10%)	10 (5.03%)		

IOL, Induction of labor.

We found significant difference in the incidence of chorioamnionitis between two groups, with 12 cases in the double-balloon group and 0 case in Dinoprostone group. The significant difference in the incidence of chorioamnionitis between the two groups were found, with 12 cases in the double-balloon group and 0 cases in the Dinoprostone group. There were four incidences of placental adhesion in the double-balloon group and it did not occur in the Dinoprostone group, although it was not statistically significant. The amount of blood loss within 24 h was higher in the Dinoprostone group, with a mean of 450 ml, while it was only 390 ml in the double-balloon catheter group (*P* < 0.0001). However, there was no occurrence of primary PPH in the Dinoprostone group. Five patients (2.51%) had primary PPH in the double-balloon catheter group, although the difference was not statistically significant (Table 4). No difference was observed in the neonatal outcomes except for birth weight and infection of the newborn. In detail, the birth weight in the double balloon catheter group was slightly higher than in the Dinoprostone group (3385.1 ± 426.0 g vs. 3297.7 ± 301.9 g, *P* = 0.0226); the number of infection of the newborn in Dinoprostone group was significantly lower than that in balloon catheter group (0 vs. 8, *P* = 0.0077, Table 5).

**Table 4:** Maternal outcome and complications during labor induction.

	**Dinoprostone (*n* = 182)**	**Double-balloon catheter (*n* = 199)**	***T*/χ^2^**	***P***
Complications (*n*, %)				
Birth canal injury	50 (27.47%)	61 (30.65%)	0.47	0.4949
Stained amniotic fluid	30 (16.48%)	47 (23.62%)	3.00	0.0832
Abnormal fetal supervision	33 (18.13%)	24 (12.06%)	2.75	0.0970^3^
Placental adhesion	0 (0.00%)	4 (2.01%)		0.1245
Chorioamnionitis	0 (0.00%)	12 (6.03%)		0.0005
Blood loss within 2 h (ml)	260 (220, 310)	300 (250, 400)	7.16	0.0075
Blood loss within 24 h (ml)	450 (390, 480)	390 (320, 530)	18.58	<0.0001
Primary PPH (*n*, %)	0	5 (2.51%)	–	0.0621

PPH, Postpartum hemorrhage.

**Table 5:** The comparison of neonatal outcomes in two groups.

	**Dinoprostone (*n* = 182)**	**Double-balloon catheter (*n* = 199)**	***T*/χ^2^**	***P***
Birth weight (g)	3297.7 ± 301.9	3385.1 ± 426.0	−2.29	0.0226
Apgar score (1 min)	9 (9, 9)	9 (9, 9)	6.3840	0.0115
Apgar score (5 min)	10 (10, 10)	10 (10, 10)	3.9247	0.0476
Fetal asphyxia (*n*, %)			–	0.0696
Without	180 (98.90%)	191 (95.98%)		
Sever	1 (0.55%)	1 (0.50%)		
Mild	1 (0.55%)	7 (3.52%)		
Inhalation syndrome (*n*, %)	4 (2.20%)	12(6.03%)	0.14	0.0753
Infection of the newborn (*n*, %)	0 (1.54%)	8 (4.02%)	–	0.0077
NICU admission (*n*, %)	4 (2.20%)	9 (4.52%)	–	0.2649
Prenatal fetal distress (*n*, %)	9 (4.95%)	10 (5.03%)	0.0013	0.9714

NICU, neonatal intensive care unit.

## Discussion

One of the most important assessment before labor induction is the Bishop score. The Bishop score is not only used to assess the cervical status, but also the chance of the success of labor induction which lead to the success of vaginal delivery (17). Low Bishop score means that the cervix is not mature enough to progress into labor, and a cervical ripening agent is needed. According to different published studies, the choice of cervical ripening agent in clinical practice depends on the Bishop score, Bishop score <4 usually uses Dinoprostone vaginal insert (15); while Bishop score ≥4 and <7 uses Cook's double balloon (16). Double-balloon catheter would be preferred more than Dinoprostone in China mostly because the Chinese obstetricians believe that occurrence of uterine hyperstimulation when using Dinoprostone is higher. This leads to the need of continuous monitoring on patients, which may lead to a serious shortage of personnel and facilities.

In 2017, the obstetricians in Hubei Province of China had evaluated the use of Dinoprostone, and revealed that the vaginal delivery rate of those using Dinoprostone was higher than that of the entire year. It was safe to use Dinoprostone in Chinese population, even in some subgroups of patients with gestational diabetes mellitus (GDM) or hypertension (18, 19). The effectiveness and safety of Dinoprostone and double-balloon catheter stratified by Bishop score were not reported. In our hospital, we have our own standard operating procedure for Dinoprostone vaginal insert patients in order to limit the incidence of uterine hyperstimulation; thus, this retrospective study was conceived. During the course of dinoprostone, a few subjects experienced temporary hypertonic uterine contractions, fetal heart rate variability, which might have lead physicians to perform a cesarean delivery (20). In China, more obstetricians prefer the balloon catheter because they believe it is safer than dinoprostone. In our study, a higher cesarean delivery rate was reported in the double balloon group compared with Dinoprostone group (24.62% vs. 9.89%, *P* = 0.0002).

Vaginal delivery can be influenced by various factors. We feel that the time required for cervical ripening was an essential factor when selecting a pre-induction strategy, and that vaginal delivery within 48 h was the most reflective of clinically relevant measures of efficacy for trials of labor induction methods. Previously, there were few studies reported the comparison between the double-balloon catheter and prostaglandin for labor induction (21). In a prospective research comparing a double-balloon catheter to Dinoprostone for IOL with an unfavorable cervix, Du et al. (22) concluded that double-balloon catheters have a comparable rate of vaginal birth within 48 h (53.9% vs. 68.4%, *P* = 0.066). In our study, the percentage of women who delivered vaginally within 48 h was considerably greater in Dinoprostone group than in double-balloon catheter group (90.11% vs. 75.38%, *P* = 0.0002). Similar to the results of the Du et al. (22) trial, the number of patients requiring oxytocin delivery was larger in double-balloon catheter group than in Dinoprostone group, indicating Dinoprostone could prolong the duration of labor.

Chorioamnionitis is a common pregnancy condition associated with adverse maternal outcomes. According to a previous study (23), the mechanical catheter was associated with a considerably higher incidence of chorioamnionitis than pharmacological labor induction. In our study, the incidence of chorioamnionitis was higher in double-balloon group than in Dinoprostone group (12 vs. 0; *P* = 0.0005), which was consistent with the findings of the previous research. Due to the injection of prostaglandin, pharmacological techniques are associated with a higher incidence of side effects, the most prevalent of which is uterine hyperstimulation. Canadas et al. showed that a vaginal delivery system for 10 mg of dinoprostone was a realistic approach for pregnant women with a high risk of uterine hyperstimulation (24). In this study, neither the Dinoprostone group nor the double-balloon catheter group experienced uterine hyperstimulation.

Postpartum hemorrhage is one of the direct causes of 30% maternal deaths all over the world (25). Previous studies have reported that the rate of PPH and the volume of PPH in cesarean section are significantly higher than that of vaginal delivery, even after labor induction by the double-balloon catheter or the Dinoprostone 10 mg vaginal insert (26). In this study, although there was no difference in the incidence of PPH between two groups, PPH occurrence in the balloon group were higher compared to the Dinoprostone group (5 vs. 0). Further, the volume of blood loss within 2 h in double-balloon catheter group were significant higher than that in Dinoprostone group. Previous study has reported that fetal weight was identified to be an accurate parameter in prediction of cesarean delivery, and increased risk of cesarean delivery and maternal complications (18). In this study, the fetal weight of the double-balloon catheter group were significant higher than that in the Dinoprostone group (3297.7 ± 301.9 vs. 3385.1 ± 426.0, *P* = 0.0226).

In general, compared with double-balloon catheter, the rate of vaginal delivery within 48 h was higher and the rate of cesarean section was lower in the Dinoprostone group, Dinoprostone could prolong the duration of labor and reduce the additional use of oxytocin during labor induction. A prospective study with larger samples is needed to verify whether Dinoprostone vaginal inserts may be safer and more efficient than double-balloon catheters to promote cervical ripening.

## Data availability statement

The raw data supporting the conclusions of this article will be made available by the authors, without undue reservation.

## Ethics statement

The studies involving human participants were reviewed and approved by the study was approved by the Ethics Committee of Zhuhai Center for Maternal and Child Health Care (Zhuhai Maternal and Child Health Hospital) (No. 2020111301). The patients/participants provided their written informed consent to participate in this study.

## Author contributions

JY and BY: conception and design. BY: administrative support. JY: provision of study materials or patients. JY and HL: collection and assembly of data, data analysis, and interpretation. HL and BY: manuscript writing. All authors contributed to the final approval of manuscript.

## Funding

This study is funded by Ferring Pharmaceuticals.

## Conflict of interest

The authors declare that the research was conducted in the absence of any commercial or financial relationships that could be construed as a potential conflict of interest.

## Publisher's note

All claims expressed in this article are solely those of the authors and do not necessarily represent those of their affiliated organizations, or those of the publisher, the editors and the reviewers. Any product that may be evaluated in this article, or claim that may be made by its manufacturer, is not guaranteed or endorsed by the publisher.
